# Norovirus shedding among symptomatic and asymptomatic employees in outbreak settings in Shanghai, China

**DOI:** 10.1186/s12879-019-4205-y

**Published:** 2019-07-08

**Authors:** Qiang-song Wu, Ze-liang Xuan, Jing-yi Liu, Xue-tao Zhao, Yuan-fang Chen, Chen-xi Wang, Xiao-ting Shen, Ya-xin Wang, Lan Wang, Yi Hu

**Affiliations:** 1 Xuhui Center for Disease Control and Prevention, Shanghai, China; 2 Xuhui District Health and Family Planning Commission, Shanghai, China; 3 Department of Epidemiology, China and Key Laboratory of Public Health Safety, (Fudan University), School of Public Health, Fudan University, Ministry of Education, No.130 Dongan Road, Xuhui District, Shanghai, 200032 China

**Keywords:** Norovirus, Acute gastroenteritis, Employees, Shedding, Asymptomatic infection

## Abstract

**Background:**

Norovirus (NoV) is recognized as a leading cause of acute gastroenteritis (AGE) outbreaks in settings globally. Studies have shown that employees played an important role in the transmission mode during some NoV outbreaks. This study aimed to investigate the prevalence of NoV infection and duration of NoV shedding among employees during NoV outbreaks, as well as factors affecting shedding duration.

**Methods:**

Specimens and epidemiological data were collected from employees who were suspected of being involved in the transmission or with AGE symptoms during NoV outbreaks in Xuhui District, Shanghai, from 2015 to 2017. Specimens were detected using real-time RT-PCR to determine whether or not employees had become infected with NoV. Specimens were collected every 3–7 days from NoV-infected employees until specimens became negative for NoV.

**Results:**

A total of 421 employees were sampled from 49 NoV outbreaks, and nearly 90% of them (377/421) were asymptomatic. Symptomatic employees showed significantly higher prevalence of NoV infection than asymptomatic ones (70.5% vs. 17.0%, *P* < 0.01). The average duration of NoV shedding was 6.9 days (95% confidence interval: 6.1–7.7 days) among 88 NoV-infected individuals, and was significantly longer in symptomatic individuals than in asymptomatic ones (9.8 days vs. 5.6 days, *P* < 0.01). In Cox proportional-hazards model, after adjusting age and gender, symptoms was the only factor associated with duration of NoV shedding.

**Conclusions:**

Compared with asymptomatic employees, symptomatic employees had higher prevalence of NoV infection and longer durations of NoV shedding. Since NoV shedding duration among NoV-infected employees tends to be longer than their isolation time during outbreaks, reinforcement of hygiene practices among these employees is especially necessary to reduce the risk of virus secondary transmissions after their return to work.

## Background

Norovirus (NoV) is one of the main pathogens causing acute gastroenteritis (AGE) in humans [1]. About 50% of AGE outbreaks worldwide are caused by NoV [2]. NoV can be transmitted by touch or through food, drinking water, or other environmental factors [3]. Since NoV spreads quickly and widely, outbreaks can easily occur in confined environments such as kindergartens, schools, hospitals, nursing homes, hotels, cruise ships, and army camps [2]. According to surveillance data from China, both the frequency of NoV outbreaks and NoV cases were rising annually from 2006 to 2013 [4], suggesting that NoV is an increasingly serious public health problem.

Studies have shown that employees such as food handlers, hospital staff, nursing home care-takers, and school teachers are involved in the transmission during NoV outbreaks [5–8]. NoV was detected in employees in 30% of NoV outbreaks in Shanghai from 2010 to 2014, and 6% of outbreaks involves NoV-infected food handlers [8]. Furthermore, nearly 30% of NoV-infected individuals were asymptomatic [9, 10]. The virus can be shed in feces or vomitus from both in symptomatic and asymptomatic NoV-infected individuals, and the duration of shedding was between 10 and 28 days [11–15]. Therefore, NoV-infected employees may become potential sources of infection after returning to work due to their persistent viral shedding.

Measures for NoV outbreak control are limited, and mainly consist of hygienic interventions and case isolation. Therefore, it is important to disseminate knowledge of NoV infection among employees to control the spread of the disease during outbreaks. To our knowledge, no studies had been reported on NoV shedding among employees during NoV outbreaks in China. Therefore, we collected specimens from employees during NoV outbreaks from 2015 to 2017, in an attempt to assess prevalence of NoV infection among employees and to investigate the possible factors affecting duration of NoV shedding.

## Methods

### Definitions

A NoV outbreak was defined as five or more epidemiologically-linked AGE cases occurring in the same setting within 3 days, of which at least two of them were laboratory confirmed as NoV infection. Symptomatic individuals were defined as the individuals who had experienced diarrhea (watery diarrhea), vomiting, abdominal pain, nausea and other AGE symptoms, which were not caused by medical treatment, food allergies, colitis, pregnancy or other causes. Asymptomatic individuals were defined as the individuals without the above AGE symptoms. NoV-infected individuals were defined as the individuals with laboratory-confirmed NoV in vomitus, feces, or anal swabs. Food handler was defined as the individuals engaged in the procurement, management, washing, cutting, crude processing, cooking, or delivery of food materials.

### Subject enrollment

**Fig. 1:**
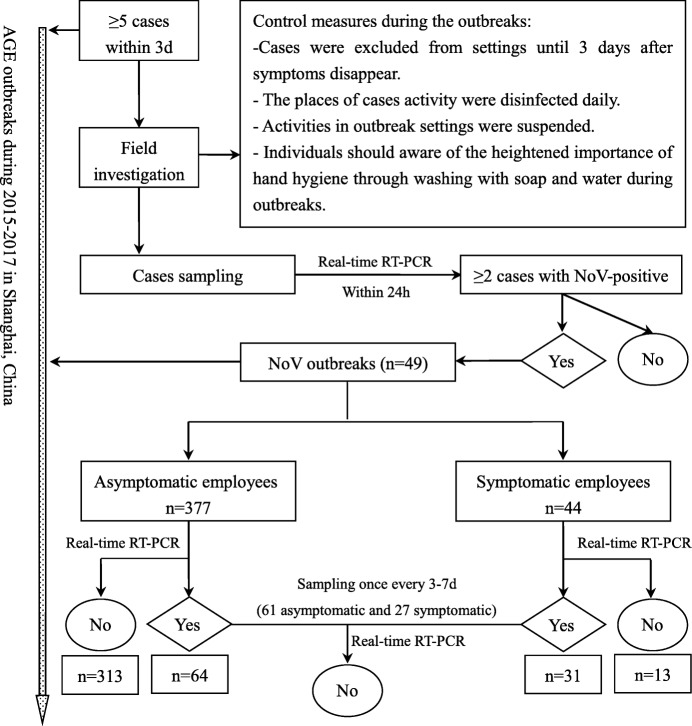
Workflow used in this study. NoV: Norovirus; AGE: acute gastroenteritis; RT-PCR: reverse transcription-polymerase chain reaction

We investigated all of the NoV outbreaks using the field epidemiological investigation method in Xuhui District, Shanghai, from 2015 to 2017. Measures for outbreak control and sampling for employees is shown in Fig. 1. Employees were enrolled for sampling during NoV outbreaks if they met any three of the following criteria: (i) employees with AGE symptoms, (ii) asymptomatic employees who were exposed to symptomatic cases, and (iii) asymptomatic employees who were suspected of being involved in the transmission during outbreaks. Specimens and epidemiological data were collected from these eligible employees including age, gender, occupation, symptoms, onset time, and sampling records.

### Sampling and NoV detection

During the outbreaks, vomitus, feces, or anal swab specimens from eligible employees were collected to detect NoV infection. The anus swabs, collected by professionals, were widely used for NoV detection due to the ability to obtain specimens more quickly during the outbreak epidemiological investigation. For the subsequent samples from NoV-infected employees, feces were easier to collect without the assistance of professionals. The minimum interval sampling time from these NoV-infected individuals was 3 days, which could be appropriately extended up to 7 days due to weekends or holidays until the subject had results converted to negative (Fig. 1).

NoV, including genogroup I (GI) and genogroup II (GII), were detected using multiple real-time RT-PCR. The primer pairs and probes were used for sample screening as reported previously [16]. RNA extraction and multiple real-time RT-PCR were conducted according to Chinese guidelines on NoV outbreak investigation, prevention and control (2015) [17].

### NoV shedding

We assumed that a NoV-negative specimen indicated that the virus level in the specimen was below the limit of detection, and defined this as the time after which the individual was no longer shedding virus. The observation time for these NoV-infected employees extended from the day the first specimen was tested positive to the day the last specimen was tested negative. Shedding duration was calculated as follows: the interval from the day the first specimen was determined to be NoV-positive to the day the last positive specimen was detected, plus the median time from the day of the last NoV-positive specimen was detected to the day of the specimen was determined to be NoV-negative.

### Statistical analysis

Differences between groups were compared using χ^2^ tests, Fisher’s exact test or Student’s t tests. The Kaplan-Meier method was used to assess whether gender, age, and symptoms were associated with the shedding duration. These three factors were further included in the Cox proportional-hazards model for multivariate analysis. Shedding duration in different quartile, including 75, 50 and 25%, was used to indicate the corresponding proportion of NoV-infected employee still shedding virus at that time. A two-sided *P* value < 0.05 was considered to be statistically significant. Statistical analysis was performed using SPSS18.0 software.

## Results

### Outbreaks

A total of 49 NoV outbreaks were laboratory confirmed from 2015 to 2017. Twenty-nine NoV outbreaks occurred in kindergartens (59.2%), 16 occurred in schools (32.7%), 2 occurred in hospitals (4.1%), and 1 occurred in nursing home (2.0%) and hotel (2.0%), respectively. The reported outbreaks were concentrated in the fourth and first quarters, which accounted for 40.8 and 34.7% of all outbreaks, respectively.

### Subjects

During the study period, a total of 421 employees were sampled from these 49 NoV outbreaks, including 236 food handlers (56.1%), 73 cleaning staffs (17.3%), 67 teachers (15.9%), 13 medical staffs (3.1%), and 32 other personnels (7.6%) (Table 1). After excluding the outbreak occurred in a hotel (190 employees), an average of 4.8 employees was sampled for each outbreak. The average age of the 421 employees was 38.8 years and 277 (65.8%) were male. Of the 421 employees, 44 (10.5%) had symptoms including diarrhea, vomiting, fever, and/or abdominal discomfort, while the remaining 377 (89.5%) were asymptomatic. Of the 44 symptomatic employees, 33 (75%) experienced diarrhea on average 3.4 times per day and 21 (47.7%) suffered vomiting up to 2.6 times a day when their symptoms were most severe (Table 1).

**Table 1:** Demographics and prevalence of norovirus infection for symptomatic and asymptomatic employees during outbreaks in Shanghai, from 2015 to 2017

Characteristics	Employees *n* = 421	Asymp^a^ *n* = 377	Asymp+ (%)^b^ *n* = 64 (17.0)	Sympt^c^ *n* = 44	Sympt+ (%)^d^ *n* = 31 (70.5)	*P* Value
Age (mean ± SD)	38.8 ± 8.5	39.0 ± 7.9	39.0 ± 7.3	37.2 ± 12.3	37.4 ± 12.3	0.18
Gender						
Male	277	249	35 (14.1)	28	19 (67.9)	< 0.01
Female	144	128	29 (22.7)	16	12 (75.0)	< 0.01
Occupation						
Food handler	236	226	45 (19.9)	10	7 (70.0)	< 0.01
Cleaning staff	73	69	9 (13.0)	4	4 (100.0)	< 0.01
Teacher	67	51	3 (5.9)	16	8 (50.0)	< 0.01
Medical staff	13	10	7 (70.0)	3	2 (66.7)	1.00
Others	32	21	0 (0.0)	11	10 (90.9)	< 0.01
Type of settings						
Kindergartens	138	126	12 (9.5)	12	10 (83.3)	< 0.01
Schools	77	68	3 (4.4)	9	2 (22.2)	0.04
Hospitals	7	4	1 (25.0)	3	3 (100.0)	0.14
Nursing home	9	8	7 (87.5)	1	0 (0.0)	0.22
Hotel	190	171	41 (24.0)	19	16 (84.2)	< 0.01
Symptoms						
Average diarrhea episodes on the most severe day (mean ± SD)	33			3.4 ± 2.3	3.3 ± 1.9	
Average vomiting episodes on the most severe day (mean ± SD)	21			2.6 ± 1.8	2.7 ± 1.9	

^a^asymptomatic employees; ^b^norovirus-infected asymptomatic employees

^c^symptomatic employees; ^d^norovirus-infected symptomatic employees

### Prevalence of NoV infection

During the study period, a total of 49 feces and 372 anus swabs were collected from 421 employees, and 95 (22.6%) were NoV-positive. The positive rate of NoV in feces was higher than that of anus swabs (36.7% vs. 20.7%, χ^2^ = 6.37, *P* = 0.01). The NoV genogroup was GII among all of these NoV-positive specimens. The prevalence of NoV infection among staff from schools, kindergartens, hotels, hospitals and nursing homes was 6.5% (5/77), 15.9% (22 /138), 30.0% (57/190), 57.1% (4/7) and 77.8% (7/9), respectively (Table 2). Medical staff had the highest prevalence of NoV infection during outbreaks (69.2%), followed by other personnel (31.3%), food handlers (22.0%), cleaning staffs (17.8%), and teachers (16.4%) (Table 2). The prevalence of NoV infection among teachers in kindergartens was higher than that of teachers in schools (22.0% vs. 7.7%), but the difference was not statistically significant (χ^2^ = 2.36, *P* = 0.13).

**Table 2:** Distribution of the prevalence of norovirus infection among different occupational staff in different type of settings

Type of setting	Outbreaks (%)	+NoV/N° Food handlers (%)	+NoV/N° Cleaning staff (%)	+NoV/N° Teachers (%)	+NoV/N° Medical staff (%)	+NoV/N° Other (%)	+NoV/N° Total (%)
Kindergartens	29 (59.2)	0/29 (0.0)	13/68 (19.1)	9/41 (22.0)			22/138 (15.9)
Schools	16 (32.7)	3/46 (6.5)	0/5 (0.0)	2/26 (7.7)			5/77 (6.5)
Hospitals	2 (4.1)				3/6 (50.0)	1/1 (100.0)	4/7 (57.1)
Nursing Home	1 (2.0)	1/2 (50.0)			6/7 (85.7)		7/9 (77.8)
Hotel	1 (2.0)	48/159 (30.2)				9/31 (29.0)	57/190 (30.0)
Total	49 (100.0)	52/236 (22.0)	13/73 (17.8)	11/67 (16.4)	9/13 (69.2)	10/32 (31.3)	95/421 (22.6)

+NoV: Norovirus-infected employees; N°: Number of employees screened for norovirus

The prevalence of NoV infection in the 44 symptomatic patients was significantly higher than that in the 377 asymptomatic employees (70.5% vs. 17.0%, χ^2^ = 64.5, *P* < 0.01). In addition to medical staff, this difference can be seen in all of other occupations (Table 1). The prevalence of NoV infection in asymptomatic employees was higher than that of symptomatic ones during a NoV outbreak in nursing home (87.5% vs. 0.0%), but there was no statistical difference between these two groups (Table 1).

### Shedding duration in NoV-infected employees

Of the 95 NoV-infected employees, 7 refused to participate in testing the duration of NoV shedding because of school holidays or job changes. A total of 126 feces and 10 anus swabs were collected from these 88 NoV-infected employees during their shedding NoV. The positive rate of NoV in feces was higher than that of anus swabs (55.6% vs. 30.0%), but there was no statistical difference between these two groups (χ^2^ = 2.43, *P* = 0.12). The average shedding duration was 6.9 days (95% *CI*: 6.1–7.7 days) in the 88 NoV-infected employees. Three quarters, 50 and 25% of NoV-infected employees still could shed NoV by their feces at day 4, 7, and 8, respectively (Table 3).

**Table 3:** Factors correlated with the duration of norovirus shedding among infected employees using the Kaplan-Meier method

Variables	Participants *n* = 88 (%)	Mean (95% CI)	Shedding duration in different quartile (days)^a^			*P* value
			25%	50%	75%	
Gender						0.42
Male	47 (53.4)	7.2 (5.9–8.4)	9	7	3	
Female	41 (46.6)	6.6 (5.7–7.4)	8	7	5	
Age						0.12
0–39	63 (71.6)	6.6 (5.7–7.4)	8	7	4	
≥40	25 (28.4)	7.7 (6.0–9.3)	9	8	5	
Symptoms ^b^						
Yes	27 (30.7)	9.8 (8.2–11.4)	13	9	7	< 0.01
No	61 (69.3)	5.6 (5.0–6.3)	8	7	3	
Total	88 (100.0)	6.9 (6.1–7.7)	8	7	4	

^a^Shedding duration in different quartile, including 75, 50 and 25%, was used to indicate the corresponding proportion of NoV-infected employee still shedding virus at that time

^b^In the Cox proportional-hazards model after adjusting age and gender (male vs. female), shedding duration was still significantly longer in symptomatic norovirus-infected employees compared with asymptomatic ones (*HR* = 3.4, 95% *CI*: 1.9–5.9, *P* < 0.01)

### Factors affecting shedding duration

**Fig. 2:**
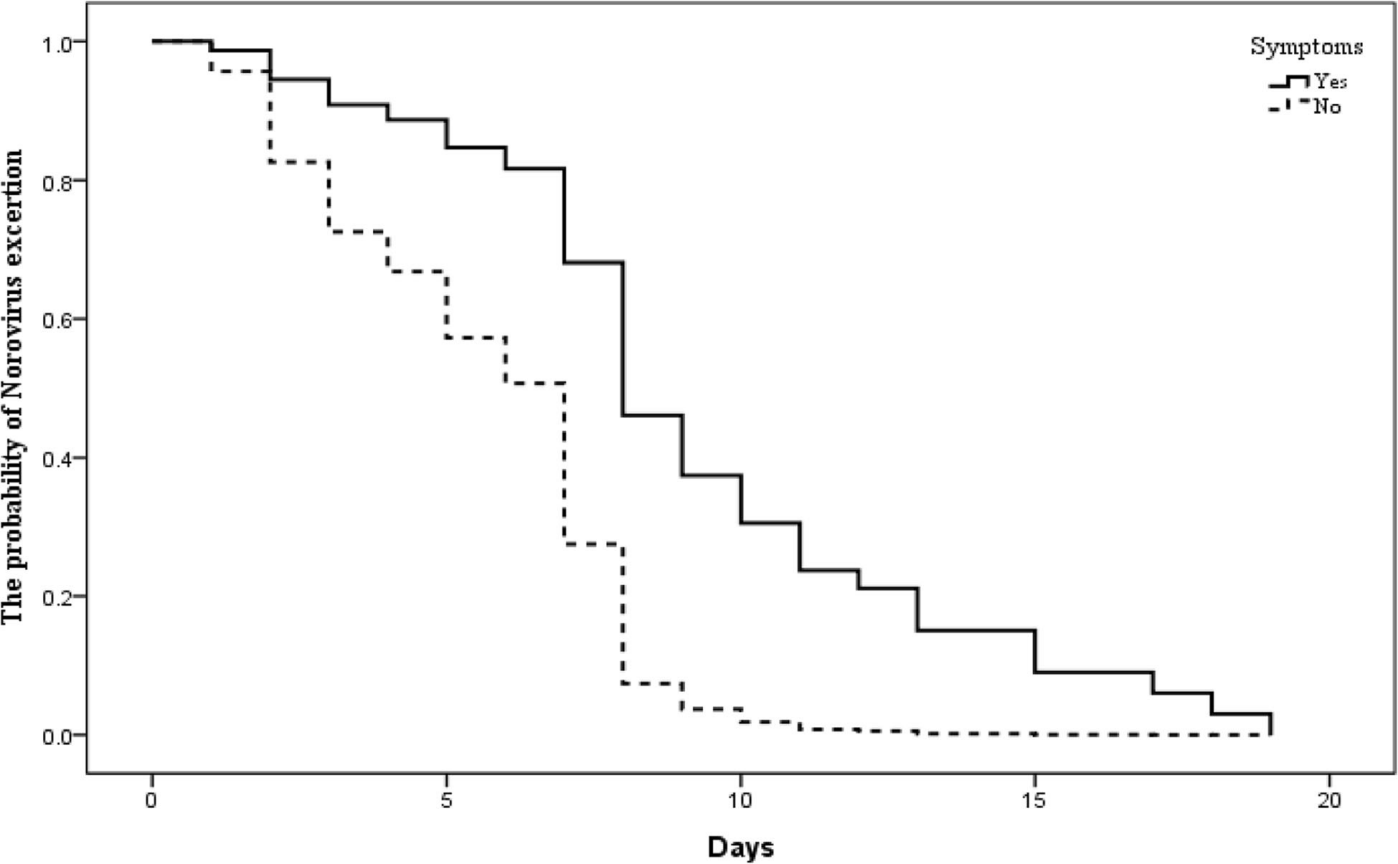
Kaplan–Meier Survival curves for association between symptom status and the duration of norovirus shedding among infected employees

There was no statistically significant difference in the NoV shedding duration between males and females (*P* = 0.42). There was also no difference in the duration of NoV detection between employees < 40 years old and those ≥40 years old (*P* = 0.12). The average duration of shedding was 9.8 days (95% *CI*: 8.2–11.4 days) in the 27 symptomatic NoV-infected individuals and 5.6 days (95% *CI*: 5.0–6.3 days) in 61 asymptomatic individuals (*P* < 0.01) (Table 3). In the Cox proportional-hazards model after adjusting age and gender, shedding duration among NoV-infected employees with no AGE symptoms was shorter (*HR* = 3.4, *P* < 0.01) (Table 3). The Kaplan-Meier curve showed that shedding duration was significantly longer in symptomatic NoV-infected employees compared with asymptomatic ones (Fig. 2).

## Discussion

In the present study, NoV was detected in over a fifth of employees during NoV outbreaks, and the prevalence of NoV infection was about four times higher in symptomatic employees than in asymptomatic ones. The prevalence of NoV infection in symptomatic employees reported here (70%) was slightly lower than that reported by Sabria et al. (77.8%) [18] but higher than that reported by Lai et al. (58.3%) [13] and Furuya et al. (29.4%) [15]. The prevalence of NoV infection in asymptomatic employees reported in our study (17%) was lower than that reported by Sabria et al. (53.7%) [18]. The differences among these findings might be explained by variation in types of NoV outbreak settings, sample size, occupation, type of specimens, and other factors. During NoV outbreaks, although the incidence of infection is significantly higher in service recipients than in employees [8], the role of employees in the spread of disease should not be neglected. Nursing staffs, teachers, and medical staffs may contribute to an “infected individual-employee-healthy individual” transmission mode during outbreaks [8], whereas food handlers may contribute to an “infected food handler-contaminated food-healthy individual” transmission mode [5, 6, 19]. Therefore, employees should be screened for NoV so as to achieve the early identification and isolation of NoV-infected individuals, no matter if they have symptoms, when they were suspected that they are involved in the transmission during outbreaks.

We found that the NoV shedding duration was significantly longer in symptomatic employees than in asymptomatic ones, which was consistent with three previous studies [13–15]. The shedding duration of symptomatic employees in our study was similar to that of NoV-infected employees in a nursing home in Taiwan (9.8 days vs. 10.0 days) [13]. Furuya et al. also observed that 76% of symptomatic inpatients and medical staff with NoV infection had shedding durations longer than 7 days [15]. However, the average NoV shedding duration of employees in our study was shorter than those reported by some studies [11–15]. These may be explained by the difference in study methods, population and its age distribution [11–15]. One systematic review study found that the duration of restriction from work after clinical symptoms for NoV cases was associated to develop gastroenteritis among residents and/or staff in nursing home during NoV outbreaks [20]. In addition, the majority of foodborne NoV outbreaks (64%) was contributed by the asymptomatic food handlers during food handling in 64 outbreaks from 2005 to 2011 [21]. Therefore, it is important to manage these NoV-infected employees who were asymptomatic or recovering but continuing to shed NoV during outbreaks. According to Chinese guidelines on NoV outbreak investigation, prevention and control (2015), isolation of both symptomatic (72 h following resolution of symptoms) and asymptomatic (72 h after testing NoV positive) NoV-infected non-food handler were recommended during outbreaks [17]. Since NoV shedding duration among NoV-infected employees tends to be longer than their isolation time, it is important to reinforce the hygiene practices among these NoV-infected employees who continue to shed virus after they return to work.

At present, larger foodborne NoV outbreaks continue to occur. As a result, the management of NoV-infected food handlers is relatively stricter than other occupations. In China, food handlers infected with NoV should be excluded until their feces or rectal swabs test negative for NoV [17]. Although negative stool results prior to returning to work for food handlers are not required in the US, they need to be approved by the regulatory authority [3]. Studies have shown that children, elderly people, and hospitalized patients tend to experience more severe symptoms after NoV infection [13, 15, 22]. Therefore, it may be worthy while to appropriate extend the isolation period for childcare staffs, nursing home staffs, and healthcare staffs when they are found to be NoV-positive. However, one study found that symptomatic patients and healthcare workers contributed most to spreading during hospital NoV outbreaks, whereas asymptomatic healthcare workers hardly transmitted virus despite high levels of fecal virus shedding [23]. At present, there is no solid evidence that the NoV continuously shed from NoV-infected individuals is infectious. Thus, it would be no good for an asymptomatic staff or those with symptoms resolved to be isolated from work even within the assumed long time of viral shedding. Fortunately, the in vitro cultivation system for human NoV, created by Ettayebi K et al. in 2016 [24], may be a good way to observe the pathogenicity of NoV that continues to be shed from NoV-infected individuals. In the future, results from the in vitro experiments will provide empirical evidence to inform prevention and control measures for NoV outbreaks.

There were some limitations to our study. Firstly, two different types of specimen (feces and anal swabs) were collected from employees in our study. The prevalence of NoV infection measured using anal swabs was significantly lower than that measured in fecal specimens in our study, which was consistent with the results of Li et al. [8]. The proportion of anal swabs among all specimens in field epidemiological investigation and NoV shedding study was 88.4 and 7.4%, respectively. Therefore, the prevalence of NoV infection among employees may be underestimated in this study, but its impact on shedding duration can’t be considered since the NoV-infected subjects shedding were followed-up mainly by the fecal sampling. Secondly, the association between initial viral load and shedding duration was not considered in this study. In the future, the real-time cycle threshold (Ct) values may be used as a proxy for viral load to assess this association. Finally, only GII type of NoV was detected and we did not consider associations between type of genogroup and shedding duration.

## Conclusions

During AGE outbreaks caused by NoV in China, NoV could be detected in over one fifth of employees. Symptomatic employees had higher prevalence of NoV infection and longer durations of NoV shedding compared with asymptomatic ones. Since NoV shedding duration among NoV-infected employees tends to be longer than their isolation time during outbreaks, reinforcement of hygiene practices among employees is especially necessary to reduce the risk of virus secondary transmissions after their return to work. More research should be done to better understand the pathogenicity of NoV that continues to be shed from NoV-infected individuals using the in vitro cultivation system in the future.

## Data Availability

The data that support the findings of this study are available from the corresponding author upon reasonable request.

## Abbreviations

AGE: Acute gastroenteritis
CI: Confidence interval
HR: Hazard ratio
NoV: Norovirus
RT-PCR: Reverse transcription-polymerase chain reaction
